# Ultrafast Process Characterization of Laser-Induced Damage in Fused Silica Using Pump-Probe Shadow Imaging Techniques

**DOI:** 10.3390/ma17040837

**Published:** 2024-02-09

**Authors:** Zhichao Liu, Jian Zhang, Shengfei Wang, Feng Geng, Qinghua Zhang, Jian Cheng, Mingjun Chen, Qiao Xu

**Affiliations:** 1 Research Center of Laser Fusion, China Academy of Engineering Physics, Mianyang 621900, China; zcliu44@163.com (Z.L.); zhangjian.leo@163.com (J.Z.); robertwsf@sina.com (S.W.); gengf0326@sina.com (F.G.); zhangqh502@sina.com (Q.Z.); 2 Center for Precision Engineering, Harbin Institute of Technology, Harbin 150001, China; cheng.826@hit.edu.cn (J.C.); chenmj@hit.edu.cn (M.C.)

**Keywords:** fused silica, laser-induced damage, ultrafast imaging, TRPP

## Abstract

This study delves into the intricate dynamics of laser-induced damage in fused silica using a time-resolved pump-probe (TRPP) shadowgraph. Three typical ultra-fast processes, laser-induced plasma evolution, shockwave propagation and material fracture splashing, were quantitatively investigated. The results indicate that the diameter of plasma is proportional to the pulse laser energy and increases linearly during the pulse laser duration with an expansion rate of approximately 6 km/s. The maximum shockwave velocity on the air side is 9 km/s, occurring at the end of the pulse duration, and then rapidly decreases due to air resistance, reaching approximately 1 km/s around a 300 ns delay. After hundreds of nanoseconds, there is a distinct particle splashing phenomenon, with the splashing particle speed distribution ranging from 0.15 km/s to 2.0 km/s. The particle sizes of the splashing particles range from 4 μm to 15 μm. Additionally, the smaller the delay, the faster the speed of the splashing particles. Overall, TRPP technology provides crucial insights into the temporal evolution of laser-induced damage in fused silica, contributing to a comprehensive understanding essential for optimizing the performance and safety of laser systems.

## 1. Introduction

Fused silica components play a crucial role in high-power laser systems. With the continuous increase in laser power, these components are susceptible to damage under intense laser irradiation, significantly affecting the laser system’s lifespan and safe operation [1]. Currently, damage to fused silica often occurs on the component’s surface, and it is widely acknowledged in the scientific community that this is closely associated with surface defects in fused silica [2,3,4]. These defects comprise two main categories: mechanical defects introduced during the fabrication process [5,6,7] and contaminative defects [8,9,10]. For a long time, researchers believed that there is a close connection between mechanical defects (e.g., cracks) and laser-induced damage [5]. It may affect damage in three ways [11,12]. First, cracks can cause discontinuities at the interface, leading to enhanced local electromagnetic fields [13,14]. Second, from a microscopic perspective, the fracture of Si-O bonds at crack sites leads to point defect enrichment, resulting in localized absorption enhancement [15,16,17]. Finally, the stress concentration at the crack tip reduces the material’s mechanical strength, promoting damage initiation and propagation [18]. Contaminative defects introduced during the polishing process are another major cause of laser-induced damage. Usually, contamination was believed to be polishing particles (e.g., CeO_2_). Impurity particles absorb laser energy through linear thermal absorption, and damage occurs when the temperature reaches the melting point. This explains the induced damage from impurity nano-particle energy absorption [19,20]. Some other researchers believe contaminants possibly originate from ion crystal particle (e.g., NaCl) precipitation during the cleaning process. This type of grain structure may contain defects, leading to intense ultraviolet absorption. Fluorescence characterization and high-damage threshold test results support this viewpoint [8]. Different types of defects exhibit distinct dynamic behaviors during the damage process, ultimately manifesting in variations in the macroscopic damage morphology and damage thresholds [21].

Optical material laser-induced damage mainly involves various laser damage mechanisms such as photoionization, avalanche ionization and impurity defect nonlinear absorption, and it can be generally categorized into intrinsic damage and extrinsic damage. Intrinsic damage mechanisms primarily address the essence of laser-induced damage without considering the thermal effects of external absorptive impurities. The damage is mainly achieved through the ionization ablation caused by the intense laser field, without involving the energy transfer process. In contrast, extrinsic damage assumes the presence of absorptive precursors, such as mechanical defects and contaminative defects. Typically, under the action of nanosecond lasers, optical component damage caused by defects falls into the category of extrinsic damage. This involves considering complex physical processes such as post-damage thermal melting, crack propagation and shock wave generation.

Therefore, investigating the dynamics of laser-induced damage serves as the foundation for understanding and, in turn, predicting damage behavior [22,23]. Laser pulses interact with materials over extremely fast time intervals, typically on the order of nanoseconds [24,25,26,27]. Conventional methods, such as high-speed cameras, face considerable challenges in capturing the evolution of defects into damage. A Time-Resolved Pump and Probe (TRPP) shadowgraph is an ultrafast imaging technique with sub-nanosecond temporal precision [28,29], well suited for probing ultrafast physical phenomena over a broad time range. This makes it particularly applicable for investigating the nanosecond laser-induced damage processes in fused silica. The technique of a TRPP shadowgraph was first applied to the study of shock waves and melting processes in silicon materials under laser irradiation by Russo R.E. et al. [30]. Around 2006, the S.G. Demos team further improved the technique and applied it to observe ultrafast physical phenomena in optical materials interacting with lasers [22,31,32]. Recently, TRPP technology achieved successful application in the characterization of transient processes in laser-induced damage, using this technique to observe important physical process such as the absorption area expansion [25], crack propagation speed [33], shock wave velocity and particle ejection [23,34,35]. It provides direct experimental evidence for the plasma explosion model [2] and absorption front theory [36].

In this study, we employed TRPP to characterize the pulse laser-induced damage process in fused silica, elucidating the evolution of the early plasma, shockwave propagation, material fracture and splashing. The obtained physical images and general patterns through TRPP contribute foundational insights into the study of laser-induced damage in fused silica.

## 2. Experimental Principles and Setup

**Figure 1:**
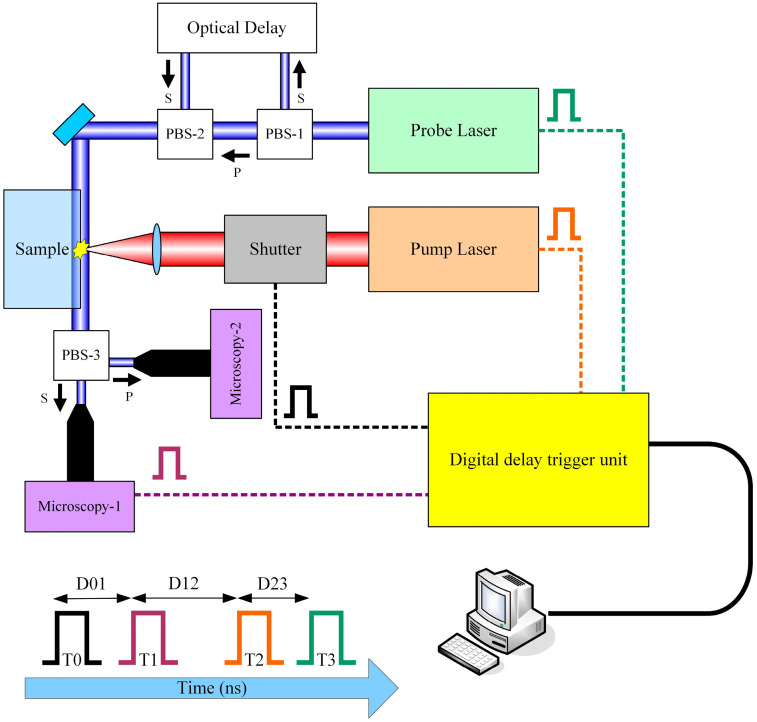
Schematic diagram of the TRPP system.

The TRPP technique is based on the pump-probe principle, and its schematic diagram is illustrated in Figure 1.

The TRPP system comprises a pump laser, a probe laser, a digital delay unit, an optical delay line, and a shadow imaging system. This study focuses on the UV laser damage of fused silica components; we chose a 355 nm wavelength nanosecond laser as the pump light source for probing the ultrafast damage process. The pump laser (wavelength of 355 nm, pulse width of 4 ns, repetition rate of 10 Hz) delivers nanosecond pulses, which are focused onto the test specimen through an optical system. By adjusting the energy of the pump laser pulses, they surpass a certain fluence threshold, inducing volumetric damage within the test specimen and triggering ultrafast physical phenomena such as plasma excitation and explosive shockwaves. Simultaneously, the probe laser (wavelength of 532 nm, pulse width of 50 ps, repetition rate of 10 Hz) emits picosecond pulses passing through the damaged region, interacting with the material in that region. Finally, the picosecond laser pulses carrying ultrafast physical information are recorded as images by the shadow imaging system. By varying the time delay between the pump and probe pulses, different time slices of the entire damage event can be captured.

To quantitatively measure transient parameters, two time slices for a single damage event need to be obtained. For this purpose, a polarization beam splitter (PBS-1 in Figure 1) is employed to split the probe laser into two beams with orthogonal polarization directions, namely, S light and P light. The relative delay between S light and P light can be adjusted by the optical delay line, as indicated by “Optical Delay” in Figure 1. PBS-2 coaxially combines S light and P light, and they pass through the damaged region together. After passing through the damaged region, the beams are separated into S light and P light by PBS-3 once again. The shadow images carrying ultrafast information are collected by dual imaging detectors. Utilizing image processing algorithms for information extraction from shadow images, transient physical quantities such as the shockwave velocity, particle ejection speed and ejection angle can be accurately calculated by precisely comparing the differences between two images. The details of the setup can be found in the reference [37].

In terms of temporal sequencing, a digital delay trigger unit (shown in Figure 1) is utilized to generate multiple channel trigger signals, T0, T1, T2 and T3, corresponding to the shutter, microscopy imaging system, pump laser and probe laser. These signals are employed for external triggering control. When the T0 trigger reaches the shutter, the shutter opens. After a delay of D01, the T1 trigger reaches the microscopic CCD, entering the exposure standby state. Following a delay of D12, the T2 trigger reaches the nanosecond laser, producing the pump irradiation pulse. After a delay of D23, the T3 trigger reaches the picosecond laser, producing the detection irradiation pulse. The delays D01, D12 and D23 are configured through control software. The triggering and capture of CCD microscopic imaging are also conducted through control software.

**Figure 2:**
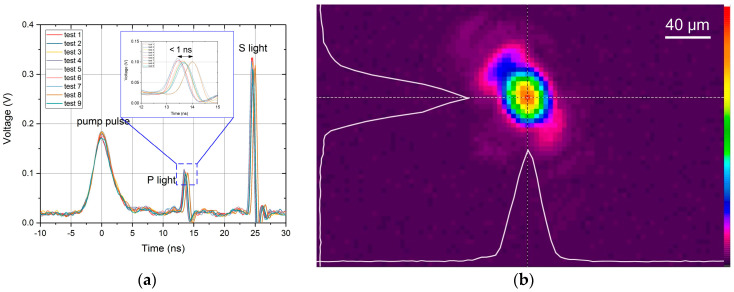
The temporal jitter error of the pump-probe pulses (**a**) and the spatial distribution of the pump laser spot at a sample’s surface under about 141 J/cm^2^ (laser energy 1.0 mJ) (**b**).

Figure 2a illustrates the pulse timing results with a pump-probe delay of 13.5 ns and an S-P light delay of 11 ns.

It can be observed that under nine repeated measurements, the system’s temporal jitter error is approximately 1 ns, fully meeting the requirements for capturing ultrafast processes in nanosecond damage. The pump laser, focused by a lens system, forms a Gaussian spot with a diameter of approximately 30 μm on the sample surface, as shown in Figure 2b. By adjusting the position of the sample displacement stage, it is easy to focus the pump laser on the front and rear surfaces of the sample.

## 3. Plasma Formation

**Figure 3:**
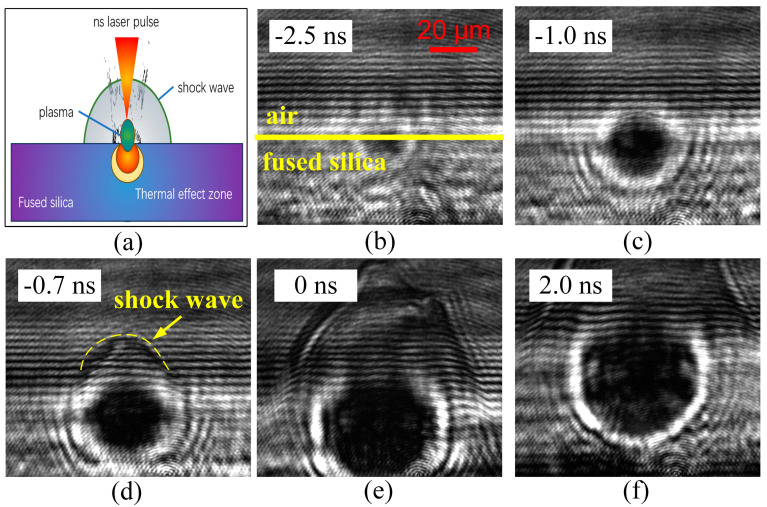
Formation and evolution process of an early plasma cluster. (**a**) schematic diagram of the interaction between laser and material, and the TRPP images under delay of −2.5 ns (**b**), −1.0 ns (**c**), −0.7 ns (**d**), 0 ns (**e**) and 2.0 ns (**f**).

Figure 3a depicts a schematic diagram of the evolution of optical material defects towards damage under the influence of nanosecond laser irradiation.

The blue substrate represents fused silica material. When the surface micro-defects are subjected to intense laser irradiation, the defects are prone to causing optical field modulation [22,23] and a decrease in material mechanical properties [38]. Additionally, considering the possible presence of thermally absorptive impurities in the defects, a large number of free electrons will be generated locally at first. The generation of free electrons rapidly increases the material’s deposition of laser energy. As the local temperature rises and the electron density increases, the surrounding material near the defect will undergo significant modification, enhancing the absorption of laser energy [24,39]. The deposition of a large amount of energy can lead to the local melting or even vaporization of the crystal in a short period, resulting in the formation of a damage pit. The vaporized material, accompanied by liquid and solid substances, is ejected from the material surface into the air, creating discontinuities in parameters such as temperature, pressure and density. Consequently, distinct shockwave phenomena can be observed in the air domain [33,34,35].

Plasma formation is the earliest phenomenon observed during the evolution from defects to damage. Under the influence of the laser field, material electrons ionize, generating free electrons. These atoms undergo collision ionization and the initial free electrons cause avalanche ionization, further increasing laser absorption and leading to the phenomenon of inverse bremsstrahlung [40,41]. The plasma cluster exhibits intense absorption, resembling metallic properties, when interacting with the probe laser. This “opaque” characteristic is precisely captured through TRPP shadow imaging. The varying shades in the images indirectly reflect the strength of probe light absorption by the plasma and are correlated with plasma density.

Figure 3b–f illustrate the ultra-early stages of plasma evolution during laser irradiation, where the yellow line segment represents the interface between the fused silica surface and the air side. Conclusions drawn from experimental observations include: 1. The plasma region forms in the early stages of the rising pump laser pulse, with a plasma cluster size of approximately 20 μm, initiating at around −2.5 ns. The initial electron density in the plasma is not high, and its absorption of the probe light is not significant. 2. With the injection of the laser pulse, the plasma volume rapidly expands within several nanoseconds, reaching its maximum at the pulse peak (in this article, we define the peak position of the pump laser pulse as the moment t = 0 delay). 3. The shockwave appears near the air side of the plasma region, occurring at approximately −1.0 ns. The diffusion velocity of the shockwave is noticeably greater than the plasma expansion velocity. Around 0.3 ns later, the shockwave separates from the plasma interface and rapidly propagates towards the air side.

From the analysis above, it is evident that plasma explosion precedes shockwave transmission and is closely related to the duration of pump laser pulse action. In contrast, the shockwave is generated early in the laser interaction process, implying that the explosion process occurs in the extremely early stages of damage.

**Figure 4:**
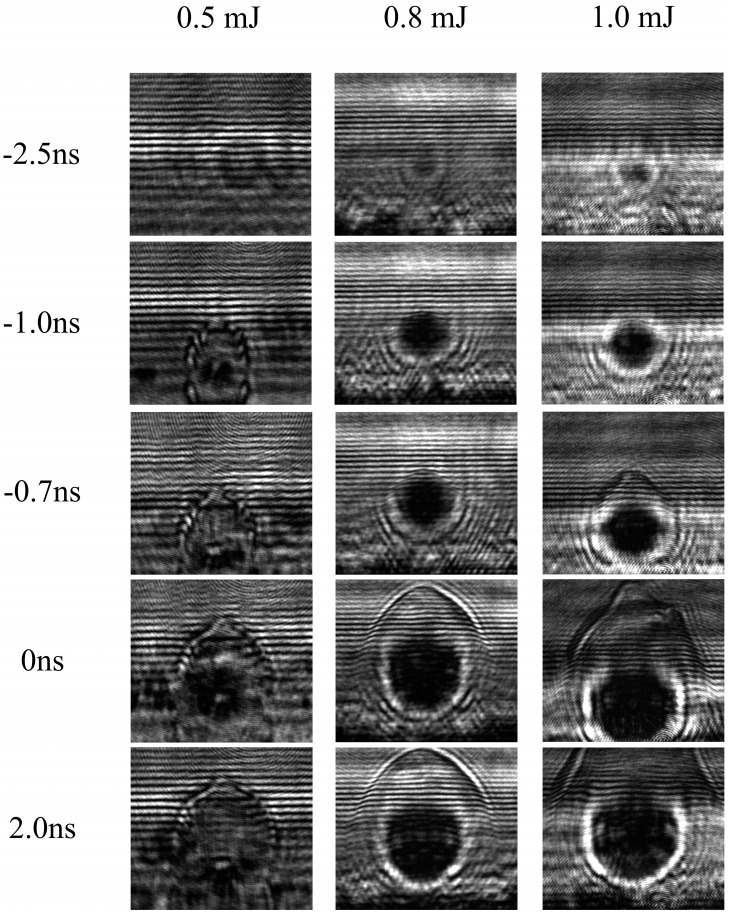
The evolution images of the plasma under different pump laser energies.

**Figure 5:**
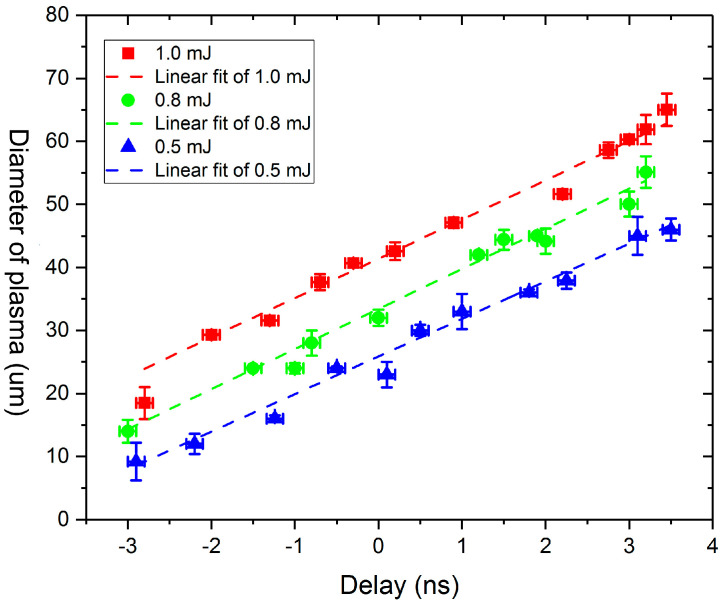
The expansion of the plasma cluster under different incident laser energies.

Utilizing image measurements, it becomes possible to precisely ascertain the expansion radius of the plasma cluster over time, thereby facilitating a comprehensive understanding of its dynamic behavior. These data serve as a crucial foundation for the subsequent determination of the expansion rate of the plasma cluster. To systematically investigate the impact of varying energy of the incident laser, Figure 4 and Figure 5 depict the expansion patterns of plasma clusters subjected to different laser excitation energies. From Figure 5, it can be observed that the plasma diameter under different laser energies varies linearly with time, with the slope representing the plasma expansion velocity, about 6 km/s.

The incident laser energies are deliberately varied, arranged in ascending order as 0.5 mJ, 0.8 mJ and 1.0 mJ, respectively. The temporal evolution of the plasma cluster expansion is captured on the horizontal axis, with the zero-moment strategically aligned with the peak position of the pump laser pulse waveform. This temporal alignment ensures a precise correlation between the observed phenomena and the temporal dynamics initiated by the laser excitation. By meticulously manipulating the energy of the incident laser, a comprehensive dataset is generated, allowing for a nuanced analysis of the expansion patterns at different excitation intensities.

The initiation of the plasma cluster becomes evident at approximately −3.0 ns, marking the inception of its formation. An intriguing observation emerges as the effective diameter of the plasma cluster, quantified as the integral of all shaded areas equivalent to the circular area, demonstrates a direct proportionality to the energy of the incident laser. This implies that the magnitude of the incident laser energy directly influences the initial diameter of the plasma cluster, with higher energy inputs resulting in larger initial diameters.

As temporal evolution unfolds, the plasma cluster embarks on a phase of rapid expansion. Remarkably, during the duration of the laser pulse, the expansion velocity of the plasma cluster remains constant. However, an interesting departure from the initial proportional relationship between the size and incident energy becomes apparent. Unlike the initial stage, wherein larger incident energies lead to larger plasma cluster diameters, the subsequent expansion phase appears to decouple from the influence of incident energy. In other words, as time progresses within the pulse duration, the size of the plasma cluster ceases to exhibit an overtly proportional relationship with the incident energy.

## 4. Shockwave Propagation

**Figure 6:**
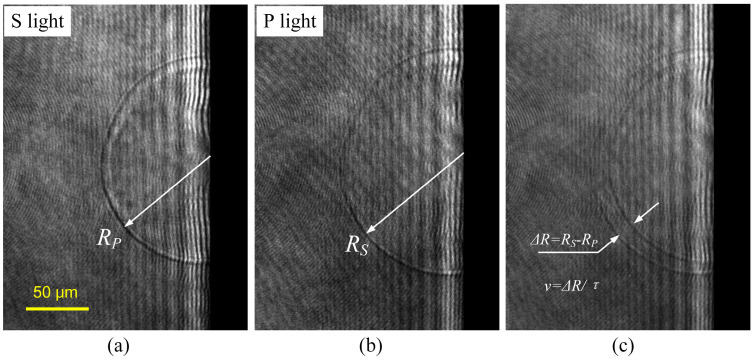
Measuring shockwave velocity using the TRPP setup. (**a**) TRPP image of S light; (**b**) TRPP image of P light; (**c**) overlap the S light image with the P light image.

The shockwave forms in the mid-term of the plasma cluster, initiating a few nanoseconds after the laser interaction and propagating simultaneously toward the optical material side and the air side. The decay behavior of the shockwave velocity directly reflects the explosive internal energy during material micro-explosions, holding significant relevance in assessing the severity of damage caused [38]. The shockwave encounters numerous neutral particles at the interface with air, resulting in a higher refractive index ahead of the shockwave compared to the background air. As light passes through the shockwave, it collapses inward, causing internal refraction. This refractive effect manifests as an externally dark and internally bright shockwave structure on the imaging plane, with the degree of shadowing proportional to the second derivative of the refractive index [42]. Therefore, TRPP shadow imaging can accurately capture the shockwave front position, as illustrated in Figure 6.

By setting an appropriate pump laser pulse energy to induce damage and generate the explosive shockwave, the shadow imaging system captures images of the shockwave under P-polarized and S-polarized light, as shown in Figure 6a,b. The propagation distance difference Δ*R* = *R*2 − *R*1 is calculated between the shockwave peaks in the two images, as depicted in Figure 6c. The instantaneous velocity of the shockwave at this delay is then determined as *v* = Δ*R*/*τ*, where *τ* is the time interval between P and S polarized light.

**Figure 7:**
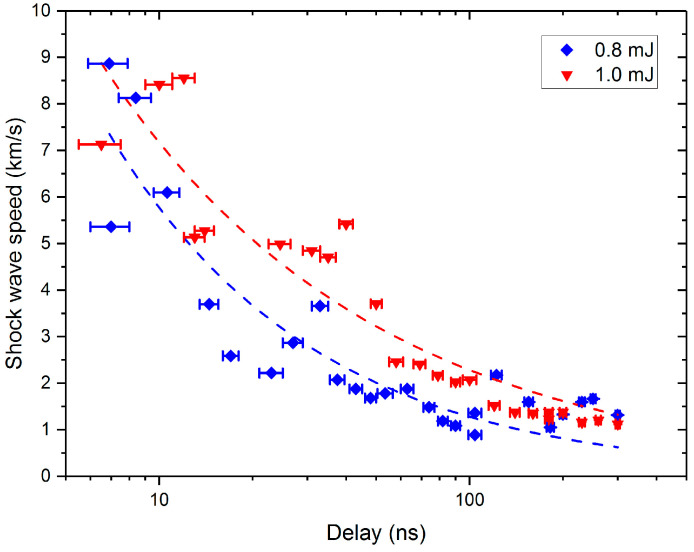
Comparison of shockwave velocities on the air side under two incident laser energies.

In the experiment, we measured the variation curve of the shockwave velocity on the air side under two different incident laser energies (1.0 mJ and 0.8 mJ), as shown in Figure 7.

It is evident that the shockwave transmission rate on the air side exhibits a clear decay pattern over time. For example, at a pump energy of 1.0 mJ, the maximum shockwave velocity of 7.1 km/s occurs at 6.4 ns, followed by a rapid decay influenced by air resistance, reaching about 1 km/s near 300 ns. The decay tendency of the shockwave velocity can be fitted using the Sedov formula [43], showing an exponential decay. Additionally, it is observed that the shockwave velocity is influenced by the incident laser energy, with higher laser energy resulting in faster shockwave velocities at the same delay time.

**Figure 8:**
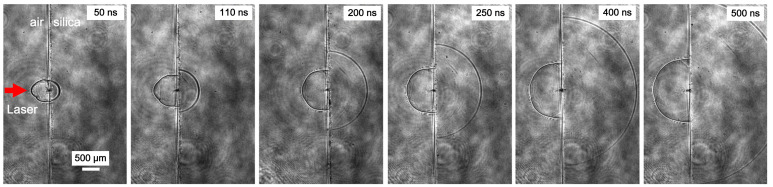
The propagation positions of the shockwaves inside the fused silica and on the air side.

The shockwave propagates concurrently in both the directions of the surrounding air and the material interface. Significantly, within the material medium, the shockwave exhibits a markedly higher velocity compared to its propagation in the adjacent air. This discrepancy leads to a rapid expansion of the spatial separation between the two shockwaves, a phenomenon vividly illustrated in Figure 8.

It is noteworthy that, in stark contrast to the swift velocity decay observed on the air side, the mechanical wave’s propagation speed penetrating the material substrate maintains a consistent value, measuring approximately 5.2 km/s. Notably, this velocity is found to surpass that experienced on the air side, contributing to the distinctive dynamic behavior exhibited within the material. This consistent and elevated velocity within the material side underscores the unique characteristics of the mechanical wave as it traverses through the medium, thereby influencing the evolving dynamics of the shockwave interaction. The asymmetry in velocity profiles between the air and material sides delineates a complex interplay of physical forces and material properties, providing valuable insights into the intricate dynamics governing the shockwave–material interaction.

## 5. Material Fracture and Splashing

In the last stage of damage, under the action of the explosive shockwave, optical materials begin to exhibit fracture splashing phenomena. The velocity, angle, and intensity of splashing are directly related to the final formation of damage. TRPP allows for the quantitative acquisition of relevant transient physical quantities.

**Figure 9:**
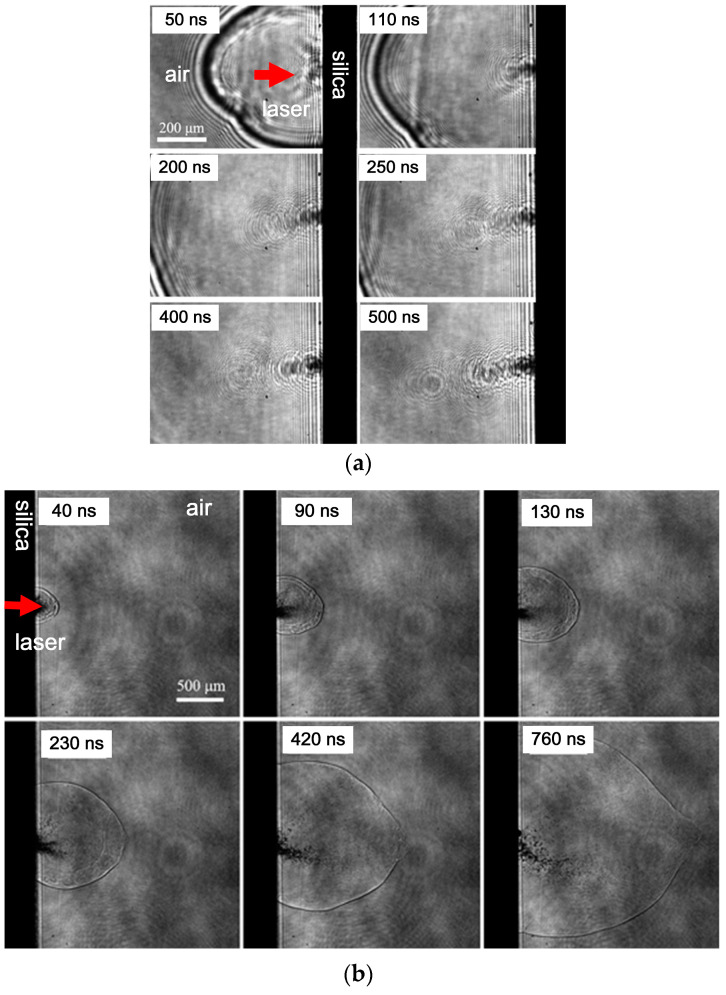
Comparison of particle splashing phenomena on the front surface (**a**) and rear surface (**b**) at different delays in the nanosecond range.

Damage can occur on the front and rear surfaces of fused silica components, and there are certain differences in particle splashing phenomena at different surface positions. Figure 9 illustrates the particle splashing process on the front and rear surfaces within the 40 ns to 760 ns delay range. Focusing the pump laser on the front surface of the sample will induce front surface damage, as shown in Figure 9a. Similarly, focusing the pump laser on the back surface of the sample will induce back surface damage, as shown in Figure 9b. In addition, the experimental parameters in Figure 9a are the same as those in Figure 8.

A comparison of the images reveals several distinctions. First, the ejection angles are different, with the rear surface ejection angle often greater than that of the front surface. The rear surface ejection angle ranges from 30° to 60°, while the front surface ejection angle is smaller (around 10°), and the ejection path is generally straight. Second, the intensity of particle splashing varies. Splashing on the front surface of fused silica begins around several tens of nanoseconds (~50 ns), with early splashing being primarily high-speed gas or nano-sized particles, making imaging unclear. In contrast, rear surface splashing is more intense, with dense and clear splashed particles.

**Figure 10:**
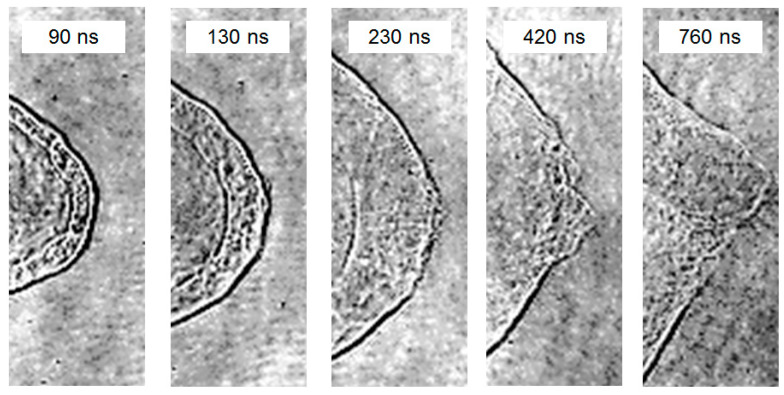
Process of the high-speed ejection of gaseous material overlapping with the shockwave front.

Further observations reveal the presence of a “cloud-like” high-speed moving substance, presumably gas-phase, within the shockwave packet. This gaseous substance rapidly expands with the shockwave, gradually exceeding the shockwave front velocity. Around 200 ns to 400 ns, it overlaps with the shockwave front and subsequently breaks through the shockwave front, creating a visible gap, as illustrated in Figure 10.

There are two possible explanations for this process. The first potential explanation is that during the explosion, two shockwaves are generated. The first and second shockwaves occur sequentially, but the velocity of the second shockwave is faster than that of the 1st, leading to their superposition at a certain moment, resulting in features resembling a shockwave front. Another possible explanation is that the shockwave propagates forward, followed by the explosion point generating high-speed ejected gas. The initial speed of the shockwave is relatively fast but quickly attenuates in the air. When the speed of the ejected gas exceeds the shockwave velocity, the two overlap, and the ejected gas breaks through the shockwave front, resulting in the phenomenon shown in Figure 10.

**Figure 11:**
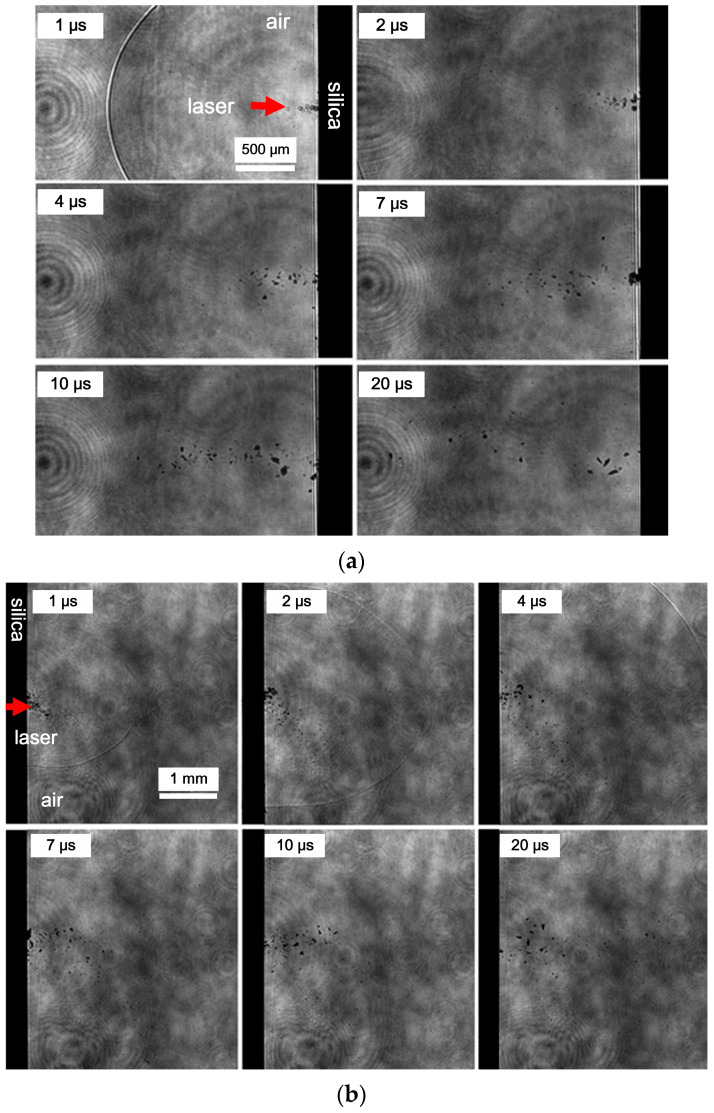
Comparison of particle splashing phenomena on the front surface (**a**) and rear surface (**b**) at different delays in the microsecond range.

As the temporal scale extends into the microsecond range, a discernible augmentation in the particle size of splashing becomes evident. This increase in particle dimensions not only enhances the quality of imaging but also facilitates the precise estimation of the particle size, elucidated in Figure 11.

During this temporal regime, both particles ejected from the front and rear surfaces manifest as irregular fragments resulting from material fracture. Notably, the disparity between the two lies in the observation that, in comparison to the front surface, the rear surface exhibits a more extensive lateral splashing range. Insightfully, researchers have noted that these irregular fragments, when meticulously collected and subjected to scanning electron microscopy (SEM) analysis, predominantly comprise material fracture fragments, with a discernibly smaller proportion displaying a melted structure [13]. This empirical observation underscores that laser-induced damage is a consequence of the synergistic interplay between high-temperature melting and mechanical fracture of the material. The intricate analysis of the ejected particles at the microsecond temporal scale provides valuable insights into the dynamic processes governing laser–material interactions, shedding light on the complex mechanisms involved in the generation of irregular fragments and melted structures during laser-induced damage events.

**Figure 12:**
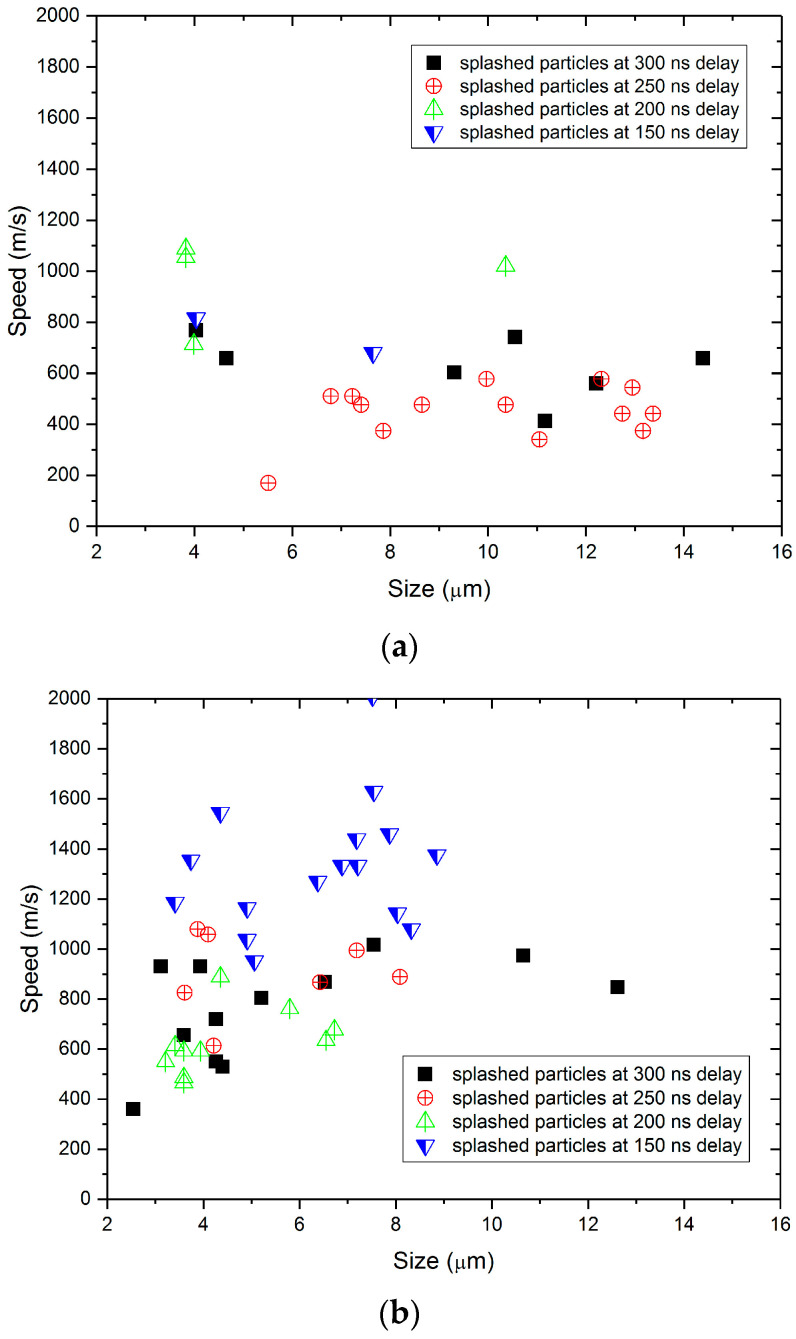
Velocity distribution of splashing particles at different laser energies: 0.8 mJ for (**a**) and 1.0 mJ for (**b**).

The method for measuring the movement speed of splashing particles is essentially the same as the method for measuring the shockwave velocity, both being based on the calculation of differences between shadow images under S/P light. Figure 12 presents the distribution of splashed particle speeds in the delay range of 150 ns to 300 ns, with Figure 12a having an incident laser energy of 0.8 mJ and Figure 12b having an incident laser energy of 1.0 mJ.

The results show that: (1) The speed distribution range of splashed particles is wide, from a minimum of 150 m/s to a maximum of 2000 m/s. (2) The smaller the delay, the faster the speed of splashed particles. (3) The incident laser energy also has a certain influence on the speed of splashed particles, with higher incident laser energy resulting in faster ejection speeds. However, there is no apparent correlation between the splashed particle size and speed.

## 6. Conclusions

In this work, we introduced the application of Time-Resolved Pump-Probe (TRPP) technology in characterizing the ultrafast processes of laser-induced damage in fused silica. We obtained some novel quantitative results that were not present in previous studies. First, we observed early plasma plumes (with delays less than 10 ns) using shadow imaging and quantitatively obtained their generation time, their expansion rate and the influence of laser energy on their evolution. The diameter of plasma is proportional to the pulse laser energy and increases linearly during the pulse laser duration with an expansion rate of approximately 6 km/s. Second, we obtained the propagation law of shock waves. After tens of nanoseconds, shockwaves begin to propagate towards the air side and into the fused silica material. The initial velocity of the shockwave is proportional to the incident laser energy. The maximum shockwave velocity on the air side is 9 km/s, occurring at the end of the pulse duration and then exponentially decreasing due to air resistance, reaching approximately 1 km/s around 300 ns of delay. Third, by comparing the differences in P/S polarization images, we have accurately obtained the velocity distribution of particle ejections in the later stage of damage. After hundreds of nanoseconds, material fracture splashing occurs in the late stage of damage, with a broad distribution of splashed particle speeds ranging from 0.15 km/s to 2.0 km/s. The particle sizes of the splashing particles range from 4 μm to 15 μm. A higher incident laser energy and shorter delay lead to faster splashed particle speeds. The smaller the delay, the faster the speed of the splashing particles. By contrasting transient images, it is observed that the ejection angle on the front surface of fused silica is smaller, with the ejection path essentially linear, while the ejection angle on the rear surface is larger, and the particle shape appears as more irregular large fragments. This difference ultimately leads to distinct damage morphologies on the front and rear surfaces.

## Data Availability

Data are contained within the article.
